# Representations of Death Among Italian Vegetarians: An Ethnographic Research on Environment, Disgust and Transcendence

**DOI:** 10.5964/ejop.v13i3.1301

**Published:** 2017-08-31

**Authors:** Ines Testoni, Tommaso Ghellar, Maddalena Rodelli, Loriana De Cataldo, Adriano Zamperini

**Affiliations:** a FISPPA Department, University of Padova, Padova, Italy; b Clinical and Social Psychologist, Padova, Italy; Webster University Geneva, Geneva, Switzerland

**Keywords:** vegetarianism, veganism, representations of death, anti-speciesism, transcendence, animal dignity, environmentalism

## Abstract

This paper focuses on the motives for vegetarian choices in contemporary Italian food culture, with specific reference to the role of the representations of death. The study adopts a qualitative research design aimed at an in-depth exploration of the reasons for avoiding meat, following an ethnographic method. Twenty-two participants (55% women, 45% men) aged 19-74, all vegetarians or vegans, mainly from Northern and Central Italy, were involved. Data from the Interpretative Phenomenological Analysis were examined according to the qualitative thematic analysis: the results show the role of death in the construction of disgust towards meat, running parallel with an emphasis on spirituality, ethical treatment of animals and the environment as reasons for avoiding meat, in particular, the concern-generating disgust and its relationship with the representation of death as a contaminating essence. The basis of disgust lies in this connection, from which the idea that oral consumption of contaminants characterized by corruptive properties, passing through the flesh of dead animals to humans, derives. The role of anti-speciesism is considered as a latent perspective, which may influence the vegetarian and vegan choices.

The importance of social factors in food choices has been thoroughly examined in various studies, exploring how motivations are prioritized in various cultures (Bisogni, Connors, Devine, & Sobal, 2002; Drewnowski, 1997; Steptoe, Pollard, & Wardle, 1995). Italy is famous for its artistic historical artefacts, and its cuisine is an important part of this cultural heritage. Indeed, food and how to cook it are very popular topics, and people of differing ages and backgrounds happily engage in conversations about food, restaurants and where to eat well. Italians are domestic tourists, precisely because food is an important feature of their travels. This interest is testified to by countless cookery magazines, inserts in newspapers and TV programs, which emphasize the diversification of regional food-related traditions. The media also represents a strong influence, on one hand, nurturing the wish to have a varied diet and try out new foods and, on the other, maintaining the importance of diversification. Tradition and home cooking still occupy a prominent place in an omnivorous diet, especially eating meat, which is one of the essential ingredients in almost all the most important recipes of main courses, which include pork, lamb, veal, mutton, poultry and game birds, and even horsemeat. Indeed, vegetarianism is still considered as the unusual choice of an eccentric minority which, however, has been recognized as passing through culinary traditions thanks to a worldwide process promoted by the mass media, which alert population to the fact that low consumption of vegetables and excessive meat are associated with diet-related diseases (Farragher, Wang, & Worsley, 2016; Wang et al., 2014). This social awareness is now an important public health goal so that vegetarianism is progressively becoming more popular, and meat-free products are more readily available (Coveney, 2006).

The denial of animal products is expressed in various forms, ranging from vegetarianism to veganism. The latter requires humans to avoid all animal products used for food, clothing or other purposes, whereas the former (“lacto-ovo vegetarianism”) allows them to consume dairy products and eggs. Today, the emergence of a vegetarian-oriented dietary style is supported by multifactor motivations related to new forms of ethical awareness, which are principles resulting from various moral viewpoints ranging from the improved quality of life to the personal sacrifice inherent in renunciation. The former belong to secular perspectives respecting healthy motivations, including personal shape and weight benefits, the dignity of animals, disgust or repugnance at eating flesh, and environmental and ecological advantages (Beardsworth & Keil, 1992; Hoek, Luning, Stafleu, & de Graaf, 2004; Povey, Wellens, & Conners, 2001; Rozin, Markwith, & Stoess, 1997; Whorton, 1994). In this sphere, “food mavenism” (competences regarding food shared among members of groups) is an important factor concerned mainly with keeping the body free of the pollution associated with meat (Somers, Worsley, & McNaughton, 2014).

Conversely, beyond the concerns inherent in the quality of food, the abstention from meat also characterizes religious ethics. Historically speaking, this kind of habit has, in fact, motivated people since ancient times (Spencer, 1993), to the extent that it remains a fundamental element of many Eastern religions, such as Hinduism, Buddhism, Jainism, Confucianism, Sikhism and Taoism (Fraser, 2002; Simoons, 1994; Walters & Portmess, 2001). In these countries, vegetarianism has been firmly established for centuries, being associated with tradition, power and status, and has inspired both religious and secularist perspectives. In Western countries, the decision to convert from a meat-eating diet is made for a range of reasons, including concerns about animal welfare, rights and martyrdom, environmental sustainability, personal health, values, gender roles, and other cultural factors (Farragher et al., 2016; Thomas, 2016; Ruby, 2012), which are intertwined with Oriental symbolisms.

This complexity confirms that vegetarianism is not just a matter of taste but rather implies the most profound dimensions, assigning significance to the existence of both individuals and the entire planet (Lindeman & Sirelius, 2001). This idea has been discussed from various perspectives, among which one is the anti-speciesism (Cohen & Regan, 2001; Regan, 2004). Anti-speciesism, which is not a widespread perspective, denounces the anthropocentric orientation and its predetermined criteria for defining the species membership, notably the domination of humankind over other animals. It tries to confute the supposed right of humans to manage the death processes (killing) of animals, considering the difficulties deriving from the fact that equality among species is impossible, as their capacities and intelligence are so variable (Cohen & Regan, 2001; Regan, 2004). From this viewpoint, the vegetarian choice is a way of respecting a different understanding of the systemic interactions between individuals, societies and the environment (Fox & Ward, 2008). Disgust can be considered an elective emotion expressing this interaction. However, the literature has not yet sufficiently stressed the latent relationship between this effect and the representations of death inherent in this alimentary style. The Rozin School proposes that disgust is functional to protect the body from eating pathogens and toxic substances (Rozin, Haidt, & McCauley, 1999). Although disgust is intimately linked to defending the body or self from contamination, it is easily extended to death-related domains. The Terror Management Theory (TMT), which emphasizes the role of the fear of death in the social construction of culture and in the construction of an individual’s values (Greenberg, Solomon, & Pyszczynski, 1997), has amply shown how disgust is fundamentally linked to reference to the animality of humans, which reminds them of their own mortality (Haidt, McCauley, & Rozin, 1994; Goldenberg et al., 2001). TMT researchers argue that many aspects of disgust help defend against the existential anxiety accompanying the realization of one's own mortality. Disgust is, therefore, a defensive emotion which maintains and emphasizes the line dividing humans, characterized by an immortal principle (the soul) and animals (mere biological flesh) (Testoni, De Cataldo, Ronconi, & Zamperini, 2017; Testoni, Parise, Visintin, Zamperini, & Ronconi, 2016). Indeed, the hierarchy between the inferiority of the finitude of flesh vs. the superiority of human thought (Wolfe, 2009) also characterizes the conflictual intergroup relationships (Haslam, 2006), where the reference to the animality of the outgroup members arouses negative emotions, disgust, rejection and violence towards such individuals (Testoni et al., 2017; Zamperini & Menegatto, 2013, 2015). Similarly, concerns about reducing the human essence to the animal state produce many bioethical discussions in the management of death and dying (Capozza, Visintin, Falvo, & Testoni, 2015; Testoni, Falletti, Visintin, Ronconi, & Zamperini, 2016). In all such complex social behaviours, the representations of death (Testoni, Ancona, & Ronconi, 2015) along with the anti-speciesism result to be particularly important.

It is possible to consider vegetarianism and veganism as behavioural expressions of the individually unknown anti-speciesist view; that means, as the result of cultural dynamics involving more and more societies and individuals in the salvation of the planet equilibrium, reducing the right of humans to kill other animals.

In this paper, we explore the ways in which dietary practices may lead to the emergence of the implicit role of life–death representations. In particular, we examine whether vegetarianism is symbolically mediated by disgust and whether this emotion ostensibly prevents us from being afraid of death. We analyse the cultural factors involved in refusing to eat meat, considering values linked to the representations of food and the body and their relationship with the representations of death and spirituality. From our point of view, vegetarianism may be considered an antispecist practice, which does not differentiate animality from humanity, but which aims at unifying these dimensions to save both of them from death. The ecological perspective assumed by vegetarians and vegans, therefore, represents the integration of the health needs of both the environment and individuals. From this perspective, the fear of death may be recognized as inherent not only to single individuals but as the connotation of an ecological ethics which expresses, through vegetarianism, the will to give a future to the entire planet, including its animals.

## Materials and Methods

This study has two main aims. One is to consider the theme of vegetarianism and veganism in its phenomenology; the other is to analyse the role of the representation of death in order to offer a new construct not yet present in the literature on this theme. Our standpoint is that vegetarianism is an anti-speciesist practice, which assimilates animality to human nature, instead of differentiating them, aimed at saving both from death. We used qualitative data in order to understand how vegetarians represent the relationships between death and the choice to live.

The literature shows that the range of vegetarianism to veganism is complex. In the opinion of Ruby (2012), there are six types of vegetarians: Type I are those who consider themselves vegetarian, yet occasionally eat red meat or poultry; Type II avoid consuming meat and poultry; Type III also avoid fish; Type IV also exclude eggs; Type V exclude dairy products produced with rennet, and Type VI (vegans) consume only vegetable-derived foods, avoiding all animal-derived food products. In our research, the inclusion criteria were the following: (a) being at least vegetarian Type II; (b) eliminating meat from daily food must have lasted uninterruptedly for at least one year; (c) speaking Italian; (d) aged at least 18 years old; (e) being motivated to participate in the research.

### Participants and Procedure

The Italian Master's course in *Death Studies and the End of Life* provided organizational support to this study by contacting several religious and health associations. An independent and trained interviewer, with no previous contacts with these associations, recruited the participants.

A total of 22 participants included 12 women (55%) and 10 men (45%), vegetarians (55%) and vegans (45%), predominantly from Northern and Central Italy, aged 19 to 74. They had eliminated animal products from their lives for a minimum of one year and a maximum of 34 years (average 7.7). Four participants were atheists and 3 agnostics; 7 defined themselves as “spiritual, but not religious” and 8 were members of a Catholic prayer community. Since people’s motivations for being vegetarian are not static and may change widely over time (Beardsworth & Keil, 1992), we asked participants to think about all the reasons they could remember why they had adopted their aim. In order to help them, we suggested some specific prefigured themes, derived both from the literature and the research aims: (a) diet description (participants were asked to provide information about their dietary choices); (b) period and context of their change in diet (when they made their vegetarian/vegan choice, i.e., the underlying reasons for their renunciation); (c) influence of diet on quality of life (in particular, whether their vegetarian/vegan diet had improved their Quality of Life); (d) spirituality, religious beliefs and belonging to religious groups; (e) representation of life and death (with participants' own definition of death and life through metaphors); (f) representation of animals' lives and death. All interviews were conducted from May to June 2016, using participant observation and Interpretative Phenomenological Analysis (IPA: Gill, 2014; Denzin, 1995) and taking part in many formal and informal meetings (food-related gatherings, spiritual and prayer meetings). After participants had signed the informed consent form, they were encouraged to talk freely; interruptions and time restrictions were avoided. Each interview lasted about 60 minutes. All participants were interviewed individually, face-to-face, while their narrations were recorded and transcribed, to be further analysed.

### Data Analysis

This study pertains to the field of qualitative research in psychology (Camic et al., 2003; Pedrazza & Berlanda, 2014), through the Interpretative Phenomenological Analysis (IPA) (Gill, 2014; Denzin, 1995) within the grounded ethnographic method (Glaser & Strauss, 1967). The combination of the emic view of the participants and the interpretative etic view of the researchers helps the understanding of cultural issues about health (Oliffe & Bottorff, 2006; Pedrazza & Boccato, 2012; Pedrazza et al., 2016; Sanjek, 2000). Following the CORE-Q check-list (Tong, Sainsbury, & Craig, 2007), our analysis is theory-driven, framing interpretations within the theories of Rozin and collaborators (1997), the TMT (Greenberg et al., 1997), and furthermore informed by anti-specist and ecologist perspectives. The object of the analysis is the interpretative repertory, which is a system of terms used to characterize and to value choices and considered as the gleaming of wider symbolic systems able to indicate the sense of avoiding meat.

Data have been analysed using the framework method for thematic qualitative analysis, which allows sources to be examined in terms of their principal concepts or themes (Marshall & Rossman, 1999). Two researchers used this approach, which is particularly appropriate, especially in ethnographic health care research (Pope, Zeibland, & Mays, 2000). The process was developed on the basis of both prior categories and categories which only became clear as analysis proceeded. The former were the basic “pre-fabricate themes” from which the latter emerged as unexpected topics. The process was divided into six main phases: preparatory organization; generation of categories or themes; coding data; testing emerging understanding; searching for alternative explanations; writing up the report (Marshall & Rossman, 1999). This method applies some of the principles of argumentation theory (Toulmin, 1958), which explores connections between explicit statements and implicit meanings of discourses (Attride-Stirling, 2001). Thematic analysis was performed with Atlas.ti, which is a software that allows us to identify thematic networks. The analysis results in network graphs, describing logical relationships between concepts and categories identified by researchers. The topic areas then form the basis for the structure of the report, within which extracts may be used to illustrate key findings.

## Results and Discussion

The analysis allowed us to highlight three dominating themes: (a) death as basis of disgust; (b) life as quality of environment; (c) spirituality of life.

### Death as the Basis of Disgust in an Individual’s Reasons for Avoiding Meat

**Figure 1:**
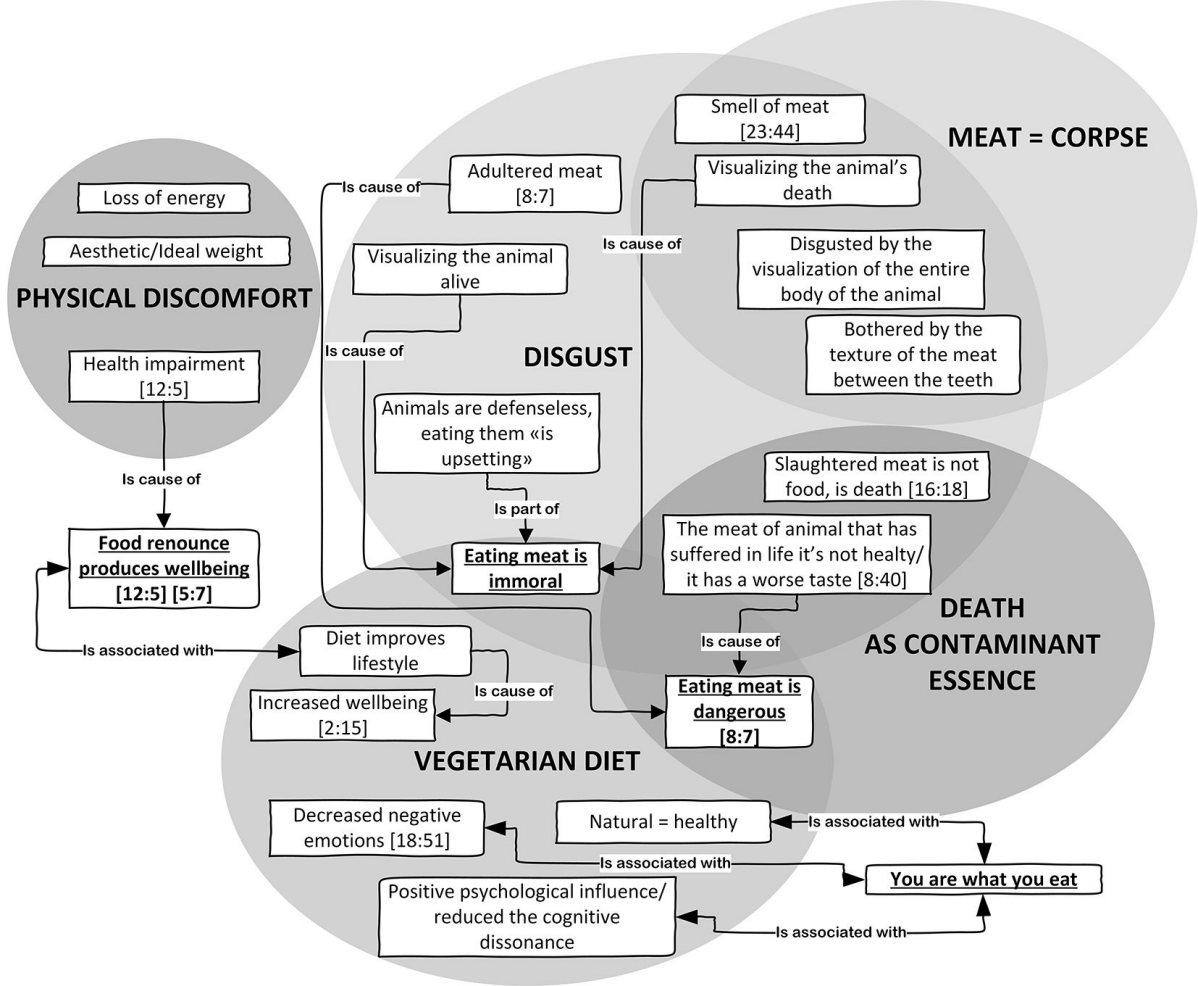
Death as the basis of disgust. *Note.* In each box of the graph, the first number in brackets indicates the quotations size (total number of quotations of the code), while the second number indicates the quotation cited in the text as examples.

At the heart of this first prevalent semantic area is the view that animals should not be mistreated since their sacrifice is also deleterious to human health (see [8:40], Table 1). The individual perspective is linked on one hand to the perception of disgust and, on the other, to the representation of health (see Figure 1).

As in Rozin et al. (1997), we collocated this first motivation factor in the area of “health vegetarianism”. In this area, dietary choices were perceived as central to good health and longevity (see [2:15], Table 1). In fact, the experience of conversion was often linked to physical unease and to a desire to become a better person, and the change to a vegetarian diet was directly linked with an improvement in health (see [12:5], [5:7], Table 1).

As emphasized by Rozin et al. (1997), the perception of disgust is an important component in the maintenance of this kind of choice (see [23:44], Table 1). Although health reasons were an initial motivator, they were also a justification for continuing to follow a meat-free diet. Animalistic ethics represents meat eating as both immoral and dangerous for human equilibrium. As described by Hamilton (2006), we observed a constant representation of the consumption of meat as a *viaticum* of contamination and sickness (see [8:7], Table 1). As reported by Fox and Ward (2008), the principle of identity - “You are what you eat” - intervenes as a crucial element in the motivation. In fact, in our research too, we found that respect for animals was associated with the belief that not consuming meat causes desirable changes in human personality and psychobiological health: (see [18:51], Table 1). Our in-depth interviews traced an essential element of this concern generating disgust: the representation of death as a contaminating essence.

The principle of corruption, which passes through meat, is death since flesh derived from dead animals is represented as part of a corpse which transmits its deadly principle and the suffering of killing (see [16:18], Table 1). The disgust derives from the oxymoron structured on the representation of animals as corpses and their state when they were alive.

**Table 1:** Health Vegetarianism

Codes	Grounded	Quotation
Adulterated meat	2	[8:7] It's also a health issue because, in fact, it is known that meat is often full of hormones to inflate it, that’s why it loses volume when you cook it, it’s because it was just full of water.
Aesthetic/Ideal weight	5	[5:2] When I was in eighth grade I started gaining weight, so our family doctor suggested that mother takes me to have some tests done and the results showed that I had thyroid problems. So I went to a centre for childhood obesity in Montebelluna, and there they found that I had an insulin resistance and thyroid problems.
Animals are defenceless, eating them “is upsetting”	2	[4:19] Most animals are defenceless creatures.
Bothered by the texture of the meat between the teeth	1	[17:5] I have never liked to eat meat because of its taste and texture. I mean, the texture of the meat between my teeth had always bothered me.
Decreased negative emotions	5	[18:51] It decreases your aggressiveness . . . and especially the agitation of the mind so that you have less violent, angry and passionate thoughts.
Disgusted by the visualization of the entire body of the animal	2	[8:3] It upsets me to clean fish, I know how to do it, but now it repels me if it is a whole fish.
Eating meat is dangerous	2	[8:7] It's also a health issue because, in fact, it is known that meat is often full of hormones to inflate it, that’s why it loses volume when you cook it, it’s because it was just full of water.
Eating meat is immoral	3	[4:19] Most animals are defenceless creatures.
Food renounce produces wellbeing	3	[5:7] I ate mainly salad for six months and my thyroid settled down.
Health impairment	3	[12:5] For example, as concerns my joint pains, I had serious problems of periarthritis and in the last years I completely resolved them with alimentation; so, when my shoulders hurt, I know that I need to further restrict my diet, that is, stop eating even dairy products and eggs, eliminate yeast and a number of other things; however, further restrictions within a vegetarian diet make you feel much better.
Improved lifestyle	4	[1:7] It has surely improved my lifestyle.
Increased wellbeing	7	[2:15] Yes, yes. No well, but I really feel much better since I do not eat meat.
Loss of energy	1	[19:42] At some point, I felt a great fatigue, a really chronic fatigue. I could not climb the stairs in my house anymore, go out, go to work, I was struggling.
Natural = healthy	6	[20:10] Changing and going back to basics and origins, with the food for excellence, that is fruit, vegetable etc, direct products from the earth.
Positive psychological influence/reduced the cognitive dissonance	5	[6:13] Well, it has become more honest! Precisely for that reason! My values coincide with what I do.
Slaughtered meat is not food, is death	1	[16:18] You can find more death than life in supermarkets. Animals brutally raised and murdered to end up in pieces, wrapped up in plastic wrap.
Smell of meat	2	[23:44] However, over the years you develop a special sensitivity, so that the idea of eating meat repels you, really even the smells, because your body is no longer accustomed to it; also the very strong smells of meat bother you.
The meat of animal that has suffered in life it's not health/it has a worse taste	2	[8:40] Because I am also sure that the mistreatment suffered by farmed animals affects the body in some way; I mean that . . . I am not able to explain it clearly, so it may seem that I am saying rubbish . . . I cannot explain you in what sense, and I am willing to think that it is just my imagination; I'm not talking about magic influences or negativity or things like that which, in my opinion are not quite tangible, but … I: So, this would affect your well-being in the end? Yes, in the end, yes! I think it's not healthy to eat the flesh of a body that has suffered in life.
Visualizing the animal alive	2	[8:11] I remember that the last time I ate meat it was hard for me to separate the emotional value from what I was doing, I mean that I was eating this piece of chicken, chicken with potatoes, a very normal thing . . . I remember exactly the physical sensation of discomfort! Because I could not stop thinking about the live animal.
Visualizing the animal’s death	1	[8:20] When I used to eat meat, I thought about the animal while he was being killed. This really bothers me.

*Note.* The table reports the codes related to the different motivations underpinning a vegetarian diet, the number of quotations identified by each code and one example of quotation for each code. The quotations are translated literally from a plain and not always correct Italian into English.

### Life Is the Quality of the Environment, and the Lack of Quality Is Death

**Figure 2:**
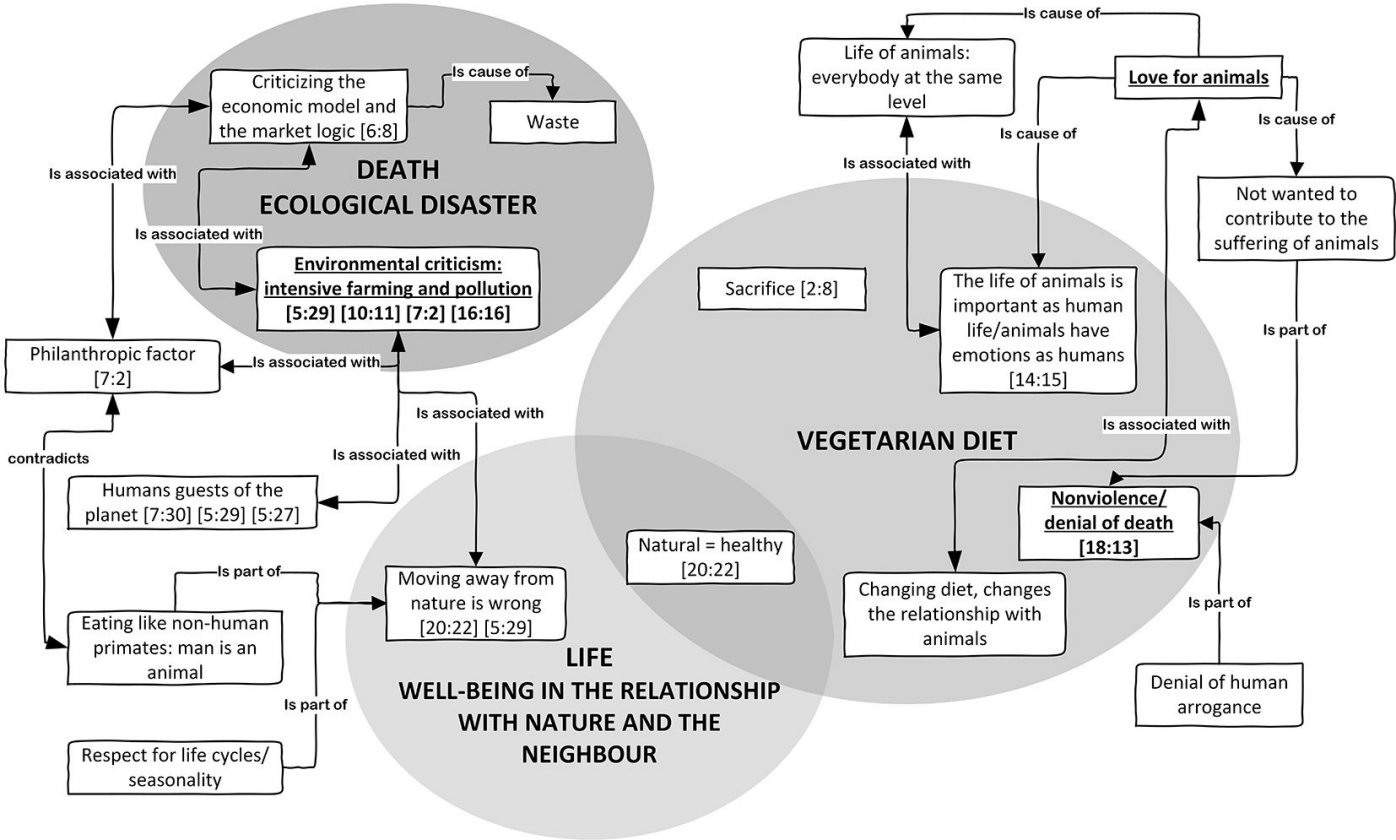
Life as environmental quality. *Note.* In each box of the graph, the first number in brackets indicates the quotations size (total number of quotations of the code), while the second number indicates the quotation cited in the text as examples.

The first reason for constructing an ecological health, which guarantees a future for the planet by limiting pollution, is the further and more transcendental basis of the vegetarian choice (Wilson, Weatherall, & Butler, 2004) (see Figure 2).

Following Rozin et al. (1997), we set this second cluster of motivations within the area of semantic prevalence inherent to “ethical vegetarianism” (Table 2). The literature shows that vegetarianism has been linked to concerns about the environmental and ecological impact of meat production (Gaard, 2002; Hoek et al., 2004; Kalof, Dietz, Stern, & Guagnano, 1999). This kind of motivation describes vegetarianism not as a mere diet but as an efficient way of confirming the validity of a broader lifestyle orientation (see [7:30], Table 2). Even though we have not found that this was the reason for refusing meat, this reason always appeared in all the narrations, assuming a tripartite form. The first component was in regard to the environmental benefits, running in parallel with personal health and spiritual advantages (see [20:22], Table 2). Many participants, whose first explanations were personal health, also described a range of environmental commitments aimed at protecting the life of the Earth (see [6:8], Table 2). In this context, vegetarianism and veganism are part of a wider view in which humans are considered as destructively affecting life on the planet (see [5:29], Table 2).

**Table 2:** Ethical Vegetarianism

Codes	Grounded	Quotation
Changing diet, changes the relationship with animals	4	[20:25] It is only over time and over the years that I started to feel there was a different relationship between me and animals, precisely because I stopped seeing them as food.
Criticizing the economic model and the market logic	9	[6:8] Well, if we have a critical approach, it is far too evident; if we do not want tropical forests to be cut down, maybe, it would be better for the world not to eat meat, because we could feed much more people with field products than with meat.
Denial of human arrogance	1	[8:37] It is not so much the fact that there is no sense, it is arrogance with no right to appeal that bothers me.
Eating like non-human primates: man is an animal	6	[19:19] If we observe non-human primates, with whom, anyhow, we share about the 98 percent of our DNA, we can see that they live on nature’s wild fruits. Otherwise, to get animal proteins, they live on small insects or, anyway, animals… I mean small animals. Anyway, their main source of food is fruit….fruit and vegetables.
Environmental criticism: intensive farming and pollution	17	[10:11] I definitely agree when people say we must eat organic stuff deriving from non-intensive farms and from the farmer.
Humans guests of the planet	5	[7:30] We are not the masters of this world, and we have no right to destroy it before both animals and people will be able to benefit from it in the future because we have abused of its resources.
Life of animals: everybody at the same level	7	[19:35] In my opinion there aren’t form of life superior to others.
Love for animals	6	[14:3] I am vegan because I fight for the animal rights. Because I love animals.
Moving away from nature is wrong	12	[5:29] If we look at what we are doing to nature, maybe we can say we are acting against it. That is, if we look at the way the world runs, we are acting pretty much the opposite way; just think about pollution or about all these factors…. And, to tell the truth, we are just acting against ourselves because nature might take revenge on us afterwards; this is not a useful way of living.
Natural = healthy	6	[20:22] We are Nature, we are an integral part of it and living in the separation we normally experience can lead only to illness.
Nonviolence/denial of death	16	[18:13] My position is not radicalism, but if someone who is defenceless must be defended, we have to do it; if justice must be defended, we have to do it, but avoiding killing is a great ideal; therefore, eating without killing is important.
Not wanting to contribute to the suffering of animals	7	[7:14] Then when I found out some more information, I saw documentaries and I discovered many other things. I said: “Ok, I do not want to contribute to this.”
Philanthropic factor	8	[7:2] I would not limit it only to that because, when I can, not only do I avoid eating animal products, foods of animal origin, and so on, but I also try to buy fair-trade foods and, now, I would also like to try to reduce waste.
Respect for life cycles and for seasonality	1	[19:8] At least, during the summer or during periods when there is plenty of fruit and vegetables; otherwise, it is more difficult during the winter because there is less variety… so I always add to my diet steam-cooked vegetables, or foods that, let's say, are a compromise, such as whole foods, brown rice, whole-grain pasta or potatoes. Even though these are cooked, let’s say they are acceptable.
Sacrifice	3	[2:8] It was not easy, not at all. As a matter of fact, I started four years ago, but with breaks, well not really breaks, with periods during which I ate meat because I felt the desire to eat it, in other words, it’s not easy! Or, maybe, you feel you have some deficiencies, you really don’t know what they are, so you start to think “I don’t know, maybe it is because I don’t eat meat”. Well, yes, this transition has been a little hard in the first years; now I would say I can do it.
The life of animals is important as human life/animals have emotions as humans	16	[14:15] Animals have different emotions as we do, and this fact is undeniable. Animals are afraid, happy, hungry and thirsty just like us. Why should their destiny be different from ours? Only because they are not protected by someone that can speak up for them, but cows and pigs are afraid and suffer just like we do.
Waste	3	[23:31] There are animals that are farmed and brutally killed so that we can eat, sometimes even excessively, really excessively. There is an excessive consumption of meat; it is also a waste, isn’t it? So, I mean, many more animals than what we can really eat are killed. And they are also reared in a brutal way.

*Note*. The table reports the codes related to the different motivations underpinning a vegetarian diet, the number of quotations identified by each code and one example of quotation for each code. The quotations are translated literally from a plain and not always correct Italian into English.

The second reason was the production and consumption of organic food seen as the final result of environmental equilibrium (see [10:11], Table 2). Control of the food production chain, which implies consumers' mavenism, is based on competence about the state of health of the Earth. Therefore, our participants indicated a number of pledges, such as reducing the use of energy and petrol for cars, limiting the amount of waste, promoting recycling, planting trees, growing vegetables in kitchen gardens, volunteering for environmental clean-ups (see “I help the earth to survive by doing a little work for the oceans”; see [16:16], [5:29], [7:2], Table 2).

The third reason was respect for animals. This element of ethical vegetarianism aims at minimizing harm to animals to be used as food or for other reasons (Fessler, Arguello, Mekdara, & Macias, 2003). The wish to avoid killing animals for human consumption was one of the main reasons given for becoming vegetarian (see [18:13], [14:15], Table 2). Both human health and animal health are part of a nexus which contributes positively to the environment: “We too should be part of nature but, at the same time, Nature must be able to live without us; in my opinion, in the end, we are just guests here” (see [5:27], Table 2). Not consuming meat is thus a sacrifice to be made by individuals as part of an ethical commitment, which includes the wellbeing of both animals and humans (see [2:8], Table 2).

### Against Death: The Spiritual Dimension of Life Is Beyond Meat and Religion

**Figure 3:**
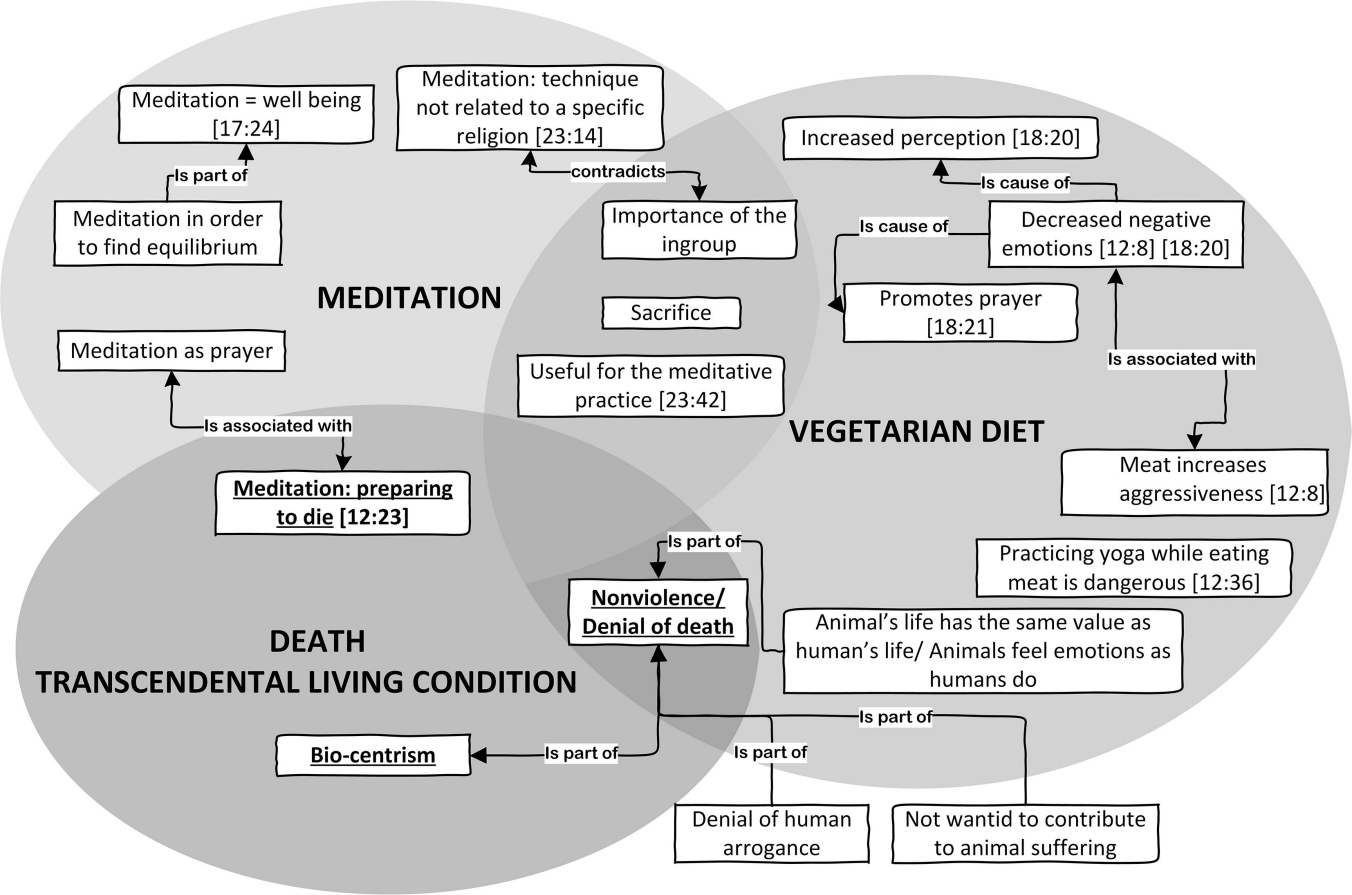
Against death. *Note.* In each box of the graph, the first number in brackets indicates the quotation size (total number of quotations of the code), while the second number indicates the quotation cited in the text as examples.

Our participants reported a wide range of issues beyond examples of diets and contributing to their existential reflection (see Figure 3).

The third dimension consists in the awareness that, in Western cultures, vegetarianism is an alternative way of life, holistically oriented by mystical practices influenced by Eastern but also universal symbolism: “From a very spiritual point of view, it helps the state of prayer, it promotes deification, it promotes the contact with the divine world because in some way we are re-assimilated to it, by eating in a way unrelated to violence and killing; it also makes your glands work in a different way, your mind works in a different way, you develop clairvoyant and sensitive abilities and prophetic qualities. Above all, meditation, which was widely practiced in early Christianity, and deep prayer are less disturbed. They are facilitated” (see [18:21], Table 3). Health often triggered these people's choice but, once adopted, eliminating meat from the diet became an aim in itself, a vector of ideological and philosophical commitment extending beyond health: “As for me, I realized it was thanks to the lifestyle choice I made, it was therefore related to meditation. In order to meditate, you have to stop eating meat because it does not help you to get deeper inside yourself”; “Everything is in relation to meditation and, in my opinion, meditation means well-being. That is, since I have been practicing it, I am much calmer. I mean, everything is functional, that's not why I do it” (see [23:42], [17:24], Table 3). Not eating meat conveyed much more than physical engagements; it also involved a plethora of cognitive, emotional, cultural and philosophical relations with concepts of purity, integrity and holism: “Eating meat, fish and taking a lot of medicines can be dangerous if you practice the headstand yoga position, blood inversion. Your blood must be quite clean”, “Meat increases aggressiveness a lot and this can be felt clearly”; “They say that, from a mental and psychological point of view, the vegetarian diet helps clarity of mind, memory, the ability to concentrate. And this was also written in ancient texts . . . even the ancient Greeks said this, and the same idea was later accepted by all the Christian fathers. It decreases your aggressiveness . . . especially agitation of the mind, so that you have fewer violent, angry, passionate thoughts, and so on” (see [24:36], [12:8], [18:20], Table 3).

**Table 3:** Spiritual Vegetarianism

Codes	Grounded	Quotations
Decreased negative emotions	5	[18:20] They say that, from a mental and psychological point of view, the vegetarian diet helps the clarity of mind, the memory, the ability to concentrate. And this is written in ancient texts eh . . . even the Ancient Greeks said this, then this same idea was picked up by all the Christian fathers afterwards. It decreases your aggressiveness . . . and especially the agitation of the mind, so that you have less violent, angry, passionate thoughts, and so on.
Denial of human arrogance	1	[8:37] It is not so much the fact that there is no sense, it is arrogance with no right to appeal that bothers me.
Importance of the ingroup	11	[23:9] As soon as I learnt about the “Ricostruttori” movement, I stopped eating meat.
Increased perception	13	[15:25] I was happy while I was walking, when I went out for a walk I had a more real perception of the world around me, like if everything was alive. The colors are brighter and the perception is more immediate.
Meat increases aggressiveness	1	[12:8] Meat greatly increases aggressiveness and this can be felt clearly, so also in this sense.
Meditation = wellbeing	3	[17:24] Everything is in relation to meditation and, in my opinion, meditation means well-being. That is, since I have been practising meditation I am much more calm. I mean, everything is functional, it is not that I do it for . . .
Meditation as prayer	9	[23:17] People consider meditation in various ways, for somebody it could be just a form of relax. In my opinion meditation is a form of prayer. And because it’s a prayer it allows you to reach the infinity, to stay with the Lord.
Meditation in order to find equilibrium	2	[11:31] I am full of contradictions, they are the engine that had led me here; they pushed me to search for something else, maybe happiness, serenity. A better balance of what I have. A strong push for change, to be better, to feel better than I am.
Meditation: preparing to die	22	[12:23] The manifestation is spirit, the spirit is in everything. In plants, in animals, in things . . . yes, reality is one, we are . . . if we were, if we knew how to vibrate as we are made for, we would be in communication with everything, with everyone and everything.
Meditation: technique not related to a specific religion	6	[23:14] Other people came to meditate with us . . . some Sufi guys, who therefore are Muslims, and until recently there was this Indian monk who stayed with us. And he also meditated with us . . . so we are very open minded. But what we are trying to do is help people just a little, to discover the spirituality existing inside everybody.
Nonviolence / Denial of death	16	[18:11] At the very beginning it was just an ethical choice. So, once they told me that you can also eat without killing animals, it made sense to me, so if you can, it is better to avoid killing.
Not wanting to contribute to animal suffering	7	[11:6] Although I liked meat, I understood it meant a lot of cruelty.
Practising yoga while eating meat is dangerous	2	[12:36] They explained to me that eating meat, fish and taking a lot of medicines can be dangerous if you practice the headstand yoga position, of the blood inversion. The blood must be rather clean. It bothered me not to go on with this things, so I decided to try.
Promotes prayer	4	[18:21] From the very spiritual point of view, it helps the state of prayer, it promotes deification, it promotes the contact with the divine world because in some way we are re-assimilated to it, by eating in a way unrelated to violence and killing; it also makes your glands work in a different way, it makes your mind work in a different way, you develop clairvoyant and sensitivity abilities, the ability to penetrate in . . . (missing audio) and prophetic qualities. And, above all, meditation, which was widely practised in early Christianity, and deep prayer are less disturbed. They are more facilitated.
Sacrifice	3	[11:11] Sometimes I feel tempted, there are things I still miss.
Useful for the meditative practice	24	[23:42] As for myself specifically, I realized it, thanks to the lifestyle choice I made, therefore related to meditation. In order to meditate you have to stop eating meat because it does not help you to go deeper inside.

*Note*. The table reports codes related to the different motivations underpinning a vegetarian diet, the number of quotations identified by each code and one example of quotation for each code. The quotations are translated literally from a plain and not always correct Italian into English.

This third level of justification explicitly translated the concept of health into that of salvation. This kind of enlightenment unified both atheists and religious believers in a common perspective, independently of faith in God: “Other people came to meditate with us . . . some Sufi friends, so they were Muslims, and until recently there was an Indian monk who stayed with us. He also meditated with us . . . so we are very open-minded. But what we are trying to do is to help people just a little, to discover the spirituality existing inside everybody” (see [23:14], Table 3). Biocentrism seems to unify these two opposite groups of vegetarians, allowing religious believers to go beyond the doctrinal perspective which considers animals as mere biological matter, without souls and thus merely as objects satisfying human desires: “The manifestation is spirit, the spirit is in everything. In plants, in animals, in things . . . yes, reality is one, we are . . . if we knew how to vibrate spiritually, as we are made for, we would be in communication with everything, with everyone and everything” (see [24:23], Table 3). These participants showed that they were aware that science demonstrated that there is no clear line separating humankind from other animals and that human exceptionalism remains central to the human sense of communal self-worth.

The perspective delineated in this research substantially seems to corroborate the idea that the concept of human uniqueness, which consists of denying animals psychological characteristics (see, secondary emotions), is a strategy of meat-eaters’ moral disengagement (Bilewicz, Imhoff, & Drogosz, 2011; Marino & Mountain, 2015). In particular, vegetarians ascribe to animals emotions and characteristics that are commonly perceived as unique to humans (Bilewicz et al., 2011) and are less likely to endorse hierarchic domination than omnivores (Allen, Wilson, Ng, & Dunne, 2000). We may, therefore, assume that vegetarians have a protectionist orientation (Blouin, 2013) characterized by bio-centric rather than anthropocentric orientation towards animals, viewing them as beings with elevated status and their own rights, and this biocentrism is spiritually linked to the idea of salvation of the Earth from death, considered as a transcendental living dimension.

### Conclusions

Among the 22 participants in this study, different initial motivations for vegetarianism were identified: personal health, disgust, animal welfare, environmentalism, and transcendence. Health was a significant motivator, both in terms of reducing symptoms of illness or discomfort and as a preventive measure to avoid a range of minor and major illnesses. Ethical reasons concerning animal welfare and the environment further motivated almost all participants for both affective and philosophical reasons, evoking transcendence and spirituality. These results show that it is impossible to reduce disgust for meat to the fear of being similar to animals. The representations of death certainly play an important role, but it is different from TMT. First, death is considered as a contaminating principle, which passes from meat to the body. Since actions are assembled and understood through a process in which different kinds of phenomena are instantiated at different levels of experience, the anti-speciesism theory may help interpretation of our results. Our participants feel as an integral part of the Earth. Therefore, they consider each other not only as individual alone but rather as a part of the environment in which the can find themselves and where they act. Much of the subject matter of motivations, such as morality and causation, relies heavily on basic metaphors derived from the experience of contact with nature growing progressively. In fact, although environmentalism is not a primary motivator for vegetarianism, it finally emerges as part of a generalization of a narrower original focus, perhaps as awareness of the sense of one's initial behaviour. As the anti-speciesism indicates, this means that not only do values and beliefs affect behaviour, but behaviour, starting from existential and moral experiences, in turn, also influences perspectives and beliefs. The representation of the environmental feedback inherent in the manipulation of animal death may imply perception on the part of the body of the return of the mortal action to the agents: killing animals and eating their flesh signifies murdering the environment and the human body as well. The behaviour of vegetarians and vegans seems to be possibly considered part of a wider moral perspective, in which humans could be represented as detrimentally affecting life on the planet. If so, dietary choices would be the first element of a concern to redress this damaging impact.

We conclude that the motivations for vegetarianism are complex, following trajectories which extend, in both terms of behaviours and values, initially adopted unwittingly, along the path of a shared reflexive awareness, becoming public. The visible component of ongoing practices, used to build the systematic action of eating, is a manifestation of a universal need for survival of which humans are an expression. Vegetarianism and veganism are becoming increasingly embedded within environmentalism although they are not in themselves the initial motivation for a meat-free diet. Indeed, from the starting point of bodily instances, additional reasons for this decision are progressively added, as individuals become increasingly complex but also more and more conscious as individuals. We may thus conclude that both ethics and the healthy behaviour of vegetarians seem to partly belong to anti-speciesist commitments, ranging from eating organic food to a variety of activities which contribute to an environmentally friendly lifestyle. Even though we cannot generalize our results because they have been obtained by idiographic methodology, we can affirm with a sufficient certainty that all this does not correspond to a mere refusal of similarity to animals but, on the contrary, to the awareness of communality deriving from sharing a common destiny.

Our findings seem to indicate that an important element of the vegetarian trajectory is the incorporation into respondents' practices and beliefs of a number of broader environmental commitments. Future research may further the exploration of ethnography as a research method to provide cultural insights for other cross-cultural differences and to locate more culturally unique food choice factors.
